# BRCA-mutated breast cancer: the unmet need, challenges and therapeutic benefits of genetic testing

**DOI:** 10.1038/s41416-024-02827-z

**Published:** 2024-08-30

**Authors:** Banu Arun, Fergus J. Couch, Jean Abraham, Nadine Tung, Peter A. Fasching

**Affiliations:** 1 Department of Clinical Cancer Genetics, University of Texas MD Anderson Cancer Center, Houston, TX USA; 2 Department of Laboratory Medicine and Pathology, Mayo Clinic, Rochester, MN USA; 3 Department of Oncology, University of Cambridge, Addenbrooke’s Hospital, Cambridge, UK; 4 Precision Breast Cancer Institute, Cambridge University Hospitals NHS Foundation Trust, Cambridge, UK; 5 NIHR Cambridge Biomedical Research Centre, Cambridge, UK; 6 Division of Hematology-Oncology, Department of Medicine, Beth Israel Deaconess Medical Center, Boston, MA USA; 7 Harvard Medical School, Boston, MA USA; 8 Department of Gynecology and Obstetrics, Comprehensive Cancer Center Erlangen-EMN, Erlangen University Hospital, Friedrich-Alexander University Erlangen-Nuremberg, Erlangen, Germany

**Keywords:** Breast cancer, Breast cancer

## Abstract

Mutations in the *BRCA1* and/or *BRCA2* genes (BRCAm) increase the risk of developing breast cancer (BC) and are found in ~5% of unselected patients with the disease. BC resulting from a germline BRCAm (gBRCAm) has distinct clinical characteristics along with increased sensitivity to DNA-damaging agents such as poly(ADP-ribose) polymerase (PARP) inhibitors and platinum-based chemotherapies, and potentially decreased sensitivity to cyclin-dependent kinase 4 and 6 (CDK4/6) inhibitors. Given the evolving treatment landscape for gBRCAm BC in early and advanced disease settings, timely determination of gBRCAm status is fundamental to facilitate the most effective treatment strategy for patients. However, many patients with gBRCAm are not identified due to suboptimal referral rates and/or a low uptake of genetic testing. We discuss current evidence for a differential response to treatment in patients with gBRCAm in early and advanced BC settings, including outcomes with PARP inhibitors, platinum-based chemotherapies, and CDK4/6 inhibitors, as well as ongoing treatment innovations and the potential of these treatment approaches. Current genetic testing strategies are also examined, including the latest guidelines on who and when to test for gBRCAm, as well as challenges to testing and how these may be overcome.

## Introduction

BRCA1 and BRCA2 proteins play critical roles in cellular DNA damage response (DDR), facilitating the conservative repair of DNA double-strand breaks as integral components of the homologous recombination repair pathway [1]. Loss-of-function mutations in breast cancer (BC) susceptibility genes 1 and 2 (*BRCA1* and *BRCA2*) can result in homologous recombination deficiency (HRD), meaning that cells are reliant on lower-fidelity repair pathways, leading to accumulation of double-strand breaks, increased genomic instability and, potentially, tumourigenesis [1]. Indeed, *BRCA1* or *BRCA2* mutations (BRCAm) can predispose an individual to develop BC, with BRCAm carriers having approximately a 70% cumulative risk of developing BC by 80 years of age [2]. BRCAm are detected in ~5% of unselected patients with BC and in ~25% of patients with a family history of BC or ovarian cancer [3].

BC resulting from germline BRCAm (gBRCAm) has distinct clinical characteristics, increased sensitivity to poly(ADP-ribose) polymerase (PARP) inhibitors and DNA-damaging agents such as platinum-based chemotherapies [1, 4], and potentially decreased sensitivity to cyclin-dependent kinase 4 and 6 (CDK4/6) inhibitors [5]. Timely determination of gBRCAm status is fundamental to establishing effective treatment strategies for patients [6]. However, many patients with gBRCAm are not identified [7–9]. Here, we discuss the evidence for differential responses in patients with early and advanced gBRCAm BC to treatments including PARP inhibitors, platinum-based chemotherapies, and CDK4/6 inhibitors, as well as ongoing treatment innovations and the potential of these treatment approaches. We also examine current genetic testing strategies and guidelines, challenges to testing, and how these challenges may be overcome.

## Brcam as an indicator of disease course and prognosis

### Overview of BRCAm breast cancer

Patients with loss-of-function mutations in *BRCA1* or *BRCA2* can present with a more aggressive BC phenotype, including triple-negative BC (TNBC) [3, 10, 11], higher tumour grade [12], and higher oncotype risk of recurrence score [13]. Patients with *BRCA1*m are more likely to develop TNBC [14], while *BRCA2*m BC can have higher nodal involvement, which is potentially associated with an increased risk of recurrence [12]. BRCAm may also influence metastatic disease burden. In PRAEGNANT, a multicentre BC registry in Germany, a higher proportion of patients with g*BRCA1*m BC had brain metastases (27.1%) compared with non-mutation carriers (12.8%); this finding was particularly marked among patients with TNBC [15]. A retrospective US study reported that the brain was a more common site of initial distant recurrence in patients with g*BRCA1*m (26.3%) than in patients with non-gBRCAm TNBC (12.1%), yet found no difference in the cumulative incidence of brain metastasis between the two populations [16].

The majority of patients with BRCAm BC present with invasive ductal carcinoma, the predominant form of BC; invasive lobular carcinoma is less common in patients with *BRCA1*m BC (~1%) than in those with *BRCA2*m BC (~7%) [10]. Up to 10% of patients with human epidermal growth factor receptor 2 (HER2)-negative BC (early or advanced) harbour a gBRCAm [3, 17]. Among HER2-negative patients, gBRCAm prevalence of >30% has been reported in some populations of patients with TNBC and ~5% among patients with hormone receptor (HR)-positive BC [3]. However, more patients with gBRCAm BC have HR-positive/HER2-negative BC, because this subtype is more prevalent than TNBC [17].

### Assessment of clinical outcomes in patients with BRCAm breast cancer

A prospective cohort study of 2733 patients with invasive BC found no significant difference in overall survival (OS) in patients with BRCAm versus those without BRCAm [12]. In addition, risk of death from breast cancer did not differ significantly between patients with and without BRCAm in a retrospective analysis of data from 1545 patients in the Israel National Cancer Registry [18]. However, it has been shown that gBRCAm positive status is a poor prognostic factor in HER2-positive BC [19]. A comprehensive meta-analysis of 35,945 patients across 30 studies observed a trend for decreased OS in patients with HR-positive/HER2-negative BC and BRCAm versus those without BRCAm, with shorter OS observed in patients with *BRCA1*m (*P* = 0.0008) and *BRCA2*m (non-significant) compared with those without BRCAm [20]. Conversely, a survival advantage has been suggested for patients with TNBC and BRCAm [12]. Although some data suggest that OS is shorter in patients with *BRCA1*m versus *BRCA2*m [21, 22], prognostic factors that are associated with each genotype (such as prevalence of TNBC) could have contributed to these observations [14]. Current evidence has not determined the full impact of BRCAm on survival outcomes across BC subtypes, and additional studies are required.

Understanding BRCAm prevalence and its impact on patients with BC has been hampered by several factors. Selection bias can arise from failure to identify all individuals with gBRCAm (since routine genetic testing is not recommended for all patients at diagnosis) [23–25], small sample sizes, and a lack of data on the BRCAm origin (i.e. germline or somatic) [10, 26, 27]. Potential confounding factors in studies examining survival outcomes include enrolment of patients at different disease stages; lack of adjustment for previous, current and subsequent treatments received; unknown clinical or pathological factors that may affect outcomes; and variation in endpoint assessments and follow-up duration [12, 20, 27]. Time-dependent differences in survival outcomes may be masked in meta-analyses that pool data from studies with different follow-up times, or from studies that do not report follow-up duration [27]. Well-designed longitudinal-outcome studies are needed to clarify the prognostic outlook for patients with BRCAm BC at all disease stages.

## Current options and future possibilities for DNA DAMAGE RESPONSE-targeted BREAST CANCER treatment

### Targeted treatment approvals for gBRCAm breast cancer

PARP inhibitors target the enzyme that plays a key role in repairing DNA single-strand breaks, uniquely exploiting synthetic lethality in HRD cells, causing replication arrest and tumour cell death. The PARP inhibitors olaparib and talazoparib are licensed for patients with gBRCAm, HER2-negative BC, in multiple disease settings [28, 29]. Olaparib is approved in the USA, Europe and Japan for the treatment of patients with gBRCAm high-risk early BC [29–31]. For locally advanced BC, olaparib is approved in Europe, and talazoparib is approved in the USA and Europe [28, 30]. Olaparib is approved for the treatment of metastatic BC in the USA, Europe and Japan, and talazoparib is approved in the USA and Europe [28–32]. Other PARP inhibitors—rucaparib, niraparib and veliparib—are not approved for BC, but have been evaluated in BC clinical trials.

### (Neo)adjuvant treatment for gBRCAm early breast cancer

#### PARP inhibitors

Several clinical trials have evaluated adjuvant or neoadjuvant PARP inhibitor treatment for gBRCAm early BC, and results are summarised in Table 1 [33–46]. Notably, the Phase III OlympiA trial demonstrated that adjuvant olaparib for 1 year, compared with placebo, could produce sustained, clinically meaningful benefits in patients with high-risk gBRCAm, HER2-negative, early BC, resulting in regulatory approval in this setting. At the primary analysis, olaparib reduced the risk of disease recurrence, with significantly longer invasive disease-free survival and distant disease-free survival versus placebo, and OS was significantly longer with olaparib than with placebo at the second survival interim analysis [34, 35]. Based on OlympiA, the American Society of Clinical Oncology, the National Comprehensive Cancer Network^®^ (NCCN^®^) and the European Society for Medical Oncology (ESMO) recommended 1 year of adjuvant olaparib for certain patients with gBRCAm, HER2-negative, early BC who are at high risk of disease recurrence after completing (neo)adjuvant chemotherapy [47–49]. Furthermore, St. Gallen International Consensus Guidelines strongly endorse adjuvant olaparib for patients with Stage II or III, HER2-negative, early BC who meet OlympiA eligibility criteria [50].

**Table 1:** Selected clinical trials of neoadjuvant and adjuvant treatments for early gBRCAm BC.

Treatment(s)	Clinical trial name/identifier	Study design	Phase	BC patient population	*N*	Selected efficacy endpoints and outcomes
Niraparib						
Neoadjuvant niraparib	NCT03329937 [33]	OL	I	gBRCAm or sBRCAm HER2-negative Resectable	21	Tumour response rate (primary endpoint): 90.5% (*n* = 19/21) pCR rate of 40% (*n* = 6/15 evaluable patients)
Olaparib ± platinum-based chemotherapy						
Adjuvant olaparib	OlympiA/ NCT02032823 [34,35]	R, DB, PC	III	gBRCAm HER2-negative Early stage	1836	Primary analysis (Year 3) Invasive disease-free survival (primary endpoint) favoured olaparib vs placebo: 85.9% vs 77.1% (hazard ratio 0.58 [99.5% CI 0.41, 0.82]; *P* < 0.001) Distant disease-free survival favoured olaparib vs placebo: 87.5% vs 80.4% (hazard ratio 0.57 [99.5% CI 0.39, 0.83]; *P* < 0.001) Key secondary analysis (OS) OS favoured olaparib vs placebo Olaparib reduced the risk of death by 32% versus placebo (hazard ratio 0.68; 98.5% CI 0.47, 0.97; *P* = 0.009): 3-year survival: 92.8% vs 89.1% 4-year survival: 89.8% vs 86.4%
Neoadjuvant olaparib + paclitaxel (OP) OR carboplatin + paclitaxel (PCb), both followed by epirubicin/cyclophosphamide	GeparOLA/ NCT02789332 [36, 37]	R, NC	II	HRD (HRD-high tumours ± gBRCAm or sBRCAm) HER2-negative Early stage	107	pCR rate (primary endpoint): no significant differences between treatment arms pCR rate for OP vs PCb: tBRCAm: 60.0% (*n* = 21/35) vs 60.0% (*n* = 12/20), *P* = 1.000 non-tBRCAm: 50.0% (*n* = 15/30) vs 37.5% (*n* = 6/16), *P* = 0.617 Invasive disease-free survival among patients with g/tBRCAm and/or HRD: hazard ratio OP to PCb, 2.86 (95% CI 0.8, 9.9) Olaparib was significantly better tolerated than carboplatin
Neoadjuvant carboplatin + paclitaxel OR carboplatin + paclitaxel + olaparib	PARTNER/ NCT03150576 [45, 46]	R, OL	II/IIII	TNBC (non-gBRCAm or gBRCAm)	762	Outcomes with carboplatin + paclitaxel + olaparib (research arm) vs carboplatin + paclitaxel (control arm) Non-gBRCAm (research arm, n = 282 vs control arm, n = 269) pCR rate: 51% vs 52% 36-month EFS rate: 80% vs 79% 36-month OS rate: 90% vs 87% gBRCAm (research arm, n = 39 vs control arm, n = 45) pCR rate: 64% vs 70% 36-month EFS rate: 96% vs 80% 36-month OS rate: 100% vs 88%
Talazoparib						
Neoadjuvant talazoparib	NEOTALA/ NCT03499353 [38]	OL	II	gBRCAm HER2-negative Early stage	61	pCR rate in the evaluable population (*n* = 48, all gBRCAm TNBC): 45.8% (*n* = 22/48)
Neoadjuvant talazoparib	NCT02282345 [39]	OL	II	gBRCAm HER2-negative Stages I–III Resectable	13	Clinical response rate: 100% (*n* = 13/13) Median reduction in tumour volume: 88% (range 30–98%)
Veliparib ± platinum-based chemotherapy						
Neoadjuvant paclitaxel + carboplatin + veliparib OR paclitaxel + carboplatin OR paclitaxel alone, all followed by doxorubicin/ cyclophosphamide	BrighTNess/ NCT02032277 [40]	R, DB, PC	III	TNBC High-risk Stages II–III	634	pCR rate in patients with BRCAm (*n* = 92): Paclitaxel + carboplatin + veliparib: 57% (*n* = 26/46) Paclitaxel + carboplatin: 50% (*n* = 12/24) Paclitaxel + placebo: 41% (*n* = 9/22)
Platinum-based chemotherapy						
Neoadjuvant cisplatin	NCT01630226 [41]	OL	II	g*BRCA1*m Stages I–III	107	pCR rate: 61% (*n* = 65/107)
Neoadjuvant carboplatin + docetaxel	PROJECT Registry/ NCT02302742, NCT01560663 [42]	OBS	NA	TNBC Stages I–III	190	pCR rate: no significant differences for gBRCAm vs non-gBRCAm (*n* = 160) 59% (*n* = 16/27) vs 56% (*n* = 75/133); *P* = 0.83
Neoadjuvant carboplatin + paclitaxel + doxorubicin + bevacizumab OR paclitaxel + doxorubicin + bevacizumab	GeparSixto (TNBC subgroup)/ NCT01426880 [43]	R	II	TNBC Stages II–III	291	pCR rate in patients with gBRCAm: no significant differences for carboplatin vs non-carboplatin gBRCAm: 65.4% (*n* = 17/26) vs 66.7% (*n* = 16/24) (odds ratio 0.94 [95% CI 0.29, 3.05]; *P* = 0.92) pCR rate in patients with non-gBRCAm favoured carboplatin vs non-carboplatin: 55.0% (*n* = 66/120) vs 36.4% (*n* = 44/121) (odds ratio 2.14 [95% CI 1.28, 3.58]; *P* = 0.004)
Neoadjuvant cisplatin OR doxorubicin + cyclophosphamide	INFORM/ NCT01670500 [44]	R	II	gBRCAm HER2-negative Stages I–III	117	pCR rate (primary endpoint): cisplatin, 18% (*n* = 11/60); doxorubicin + cyclophosphamide, 26% (*n* = 15/57) (RR 0.70 [90% CI 0.39, 1.2])

*BC* breast cancer, *BRCAm* breast cancer gene mutation, *CI* confidence interval, *DB* double-blind, *g/tBRCAm* germline or tumour breast cancer gene mutation, *EFS* event-free survival, *HER2* human epidermal growth factor receptor 2, *HRD* homologous recombination deficiency, *NA* not applicable, *NC* non-comparative, *OBS* observational, *OL* open-label, *OP* olaparib + paclitaxel, *OS* overall survival, *PC* placebo-controlled, *PCb* carboplatin + paclitaxel, *pCR* pathological complete response, *R* randomised, *RR* risk ratio, *sBRCAm* somatic BRCA gene mutation, *TNBC* triple-negative breast cancer.

Clinical trials have evaluated single-agent PARP inhibitors in the neoadjuvant setting (Table 1). A pilot study of neoadjuvant talazoparib in patients with gBRCAm BC, demonstrated a decrease in tumour volume after 2 months [39], and a modified Phase II trial demonstrated a pathological complete response (pCR) rate of 45.8% in evaluable patients with TNBC after 6 months of talazoparib [38]. Another Phase I pilot study demonstrated a tumour response rate (≥30% reduction from baseline) of 90.5% after 2 months of neoadjuvant niraparib, and a pCR rate of 40.0% in patients with BRCAm BC [33]. However, one study has reported that pCR rate was a weak predictor of prognosis in patients with gBRCAm TNBC who received neoadjuvant chemotherapy with or without bevacizumab [51].

Combining PARP inhibitors with neoadjuvant chemotherapy may maximise response rates, but toxicities have led it to be met with clinical challenges. Several studies have evaluated currently available neoadjuvant PARP inhibitors at levels below full dose in combination with chemotherapy (Table 1), with limited success. In the non-comparative Phase II GeparOLA trial, a reduced dose of olaparib (200 mg/day) plus paclitaxel versus carboplatin plus paclitaxel, both followed by epirubicin and cyclophosphamide prior to surgery, was evaluated in patients with HER2-negative, HRD tumours [36]. While pCR rates were numerically higher, and tolerability was improved with olaparib–paclitaxel versus carboplatin–paclitaxel [36], long-term analysis failed to show survival benefit with olaparib–paclitaxel in the overall population. pCR and invasive disease-free survival rates were comparable between treatment arms in the subset of patients with BRCAm. However, efficacy data from this study may have been affected by variations in post-surgery treatment [37]. The primary analysis of the Phase III neoadjuvant trial (BrighTNess) failed to demonstrate a higher pCR rate with the addition of a reduced dose of veliparib (100 mg/day) to carboplatin–paclitaxel versus carboplatin–paclitaxel alone, each arm followed by doxorubicin and cyclophosphamide, for patients with Stage II–III, high-risk TNBC [40]. In the subset of patients with gBRCAm, pCR rates were 57% with the veliparib combination and 50% with the carboplatin–paclitaxel backbone [40]; this benefit among the gBRCAm subset was not supported by a later exploratory secondary analysis using well-matched cohorts with either treatment regimen [52]. Veliparib is known to have relatively lower PARP-trapping potency compared with other PARP inhibitors, limiting extrapolation of these findings beyond veliparib [52]. A Phase II/III randomised trial (PARTNER) similarly evaluated the addition of olaparib to paclitaxel and carboplatin as neoadjuvant therapy for patients with TNBC with or without gBRCAm. Although neoadjuvant olaparib did not improve pCR rates, event-free survival (EFS) or OS compared with paclitaxel and carboplatin alone among patients with non-gBRCAm and TNBC [46], improvements in EFS and OS were observed among patients with gBRCAm who received a gap schedule of olaparib with paclitaxel and carboplatin [45]. There remains a need to optimise PARP inhibitor regimens to allow full-dose combinations in the neoadjuvant setting and further enhance outcomes.

#### Platinum-based chemotherapy

The clinical value of adding platinum therapy to neoadjuvant chemotherapy for patients with gBRCAm tumours is inconclusive (Table 1). A meta-analysis of neoadjuvant regimens in patients with gBRCAm TNBC found that pCR rates were improved when platin derivatives were combined with anthracyclines and taxanes, although it was unclear if this combination offered a clinically meaningful benefit over standard chemotherapy alone [53]. Secondary analysis of data from BrighTNess reported that pCR benefit from the addition of carboplatin to standard neoadjuvant chemotherapy in patients with TNBC was not dependent on gBRCAm status [52]. Findings from the Phase II GeparSixto and INFORM trials suggest that platinum-based chemotherapy does not provide meaningful pCR benefits over non-platinum-based regimens in patients with gBRCAm, early BC (Table 1) [43, 44]. Addition of platinum to the treatment regimen did not significantly improve disease-free survival rates in patients with BRCAm in GeparSixto [43]. Of note, a limitation of INFORM was the use of a suboptimal chemotherapy regimen, which resulted in lower pCR rates than reported in other studies [54]. Additionally, the limited number of patients with gBRCAm in GeparSixto may have impacted the observed effect of platinum-based therapy [43].

#### Surgery

Breast-conserving surgery is the preferred surgical option for most patients with early BC. However, whether a more aggressive approach (i.e. unilateral or bilateral mastectomy) is beneficial for patients with a confirmed gBRCAm tumour has been the subject of much research. A meta-analysis for risk of ipsilateral tumours following breast-conserving surgery showed a higher risk of new primary cancers for BRCAm carriers than for non-BRCAm carriers [55]. However, no differences in OS or distant recurrence were found for breast-conserving surgery versus mastectomy [55, 56]. NCCN Clinical Practice Guidelines in Oncology (NCCN Guidelines^®^) recommend that younger (≤35 years) or premenopausal patients with BRCAm early BC consider additional risk-reduction strategies in consultation with their care team, taking the risk of contralateral BC, as well as the risk of recurrence of the primary tumour, into account [47]. American Society of Clinical Oncology guidelines recommend that breast-conserving therapy be considered for patients with gBRCAm early BC while accounting for the risk of contralateral BC, the risk of recurrence of the primary tumour, and the ability of the patient to undergo continued breast surveillance with annual mammogram and magnetic resonance imaging [57].

#### Immunotherapy

Following positive results from the Phase III KEYNOTE-522 clinical trial, pembrolizumab was approved in the USA and Europe for neoadjuvant treatment in combination with chemotherapy and subsequent single-agent adjuvant treatment of patients with high-risk early-stage TNBC [58, 59]. However, no data from KEYNOTE-522 have been released comparing the clinical benefit of (neo)adjuvant pembrolizumab in patient subgroups with gBRCAm versus non-gBRCAm, and further investigation is needed to guide the most appropriate (neo)adjuvant treatment for patients with gBRCAm early BC [46].

### Treatment for locally advanced or metastatic, gBRCAm, HER2-negative breast cancer

#### PARP inhibitors

Approvals of monotherapy with the PARP inhibitors olaparib and talazoparib were based on data from OlympiAD and EMBRACA, respectively, which were Phase III, open-label, randomised, multicentre, international studies that compared PARP inhibitor monotherapy with single-agent standard therapy of the physician’s choice (TPC) [60–67]. The design and main findings from OlympiAD, EMBRACA and other key trials of PARP inhibitors are shown in Table 2 [60–66, 68–76] and have been comprehensively reviewed by Cortesi et al [77]. In both OlympiAD and EMBRACA, median progression-free survival (PFS) was significantly longer with PARP inhibitor treatment versus TPC, and was consistent across a range of patient subgroups [60, 64]. No significant differences were reported in median OS with olaparib or talazoparib versus TPC, although patients in both studies who discontinued study treatment subsequently received other medications, which likely confounded OS data [63, 66]. A study of talazoparib in the USA showed that real-world clinical outcomes were consistent with EMBRACA results [78]. Of note, an exploratory subgroup analysis of OlympiAD indicated a greater OS benefit with olaparib in the first-line setting compared with TPC, suggesting a meaningful OS benefit in patients who had not received chemotherapy for metastatic disease [63, 79]. Additional analysis of OlympiAD found that benefit with olaparib was consistent across patients stratified by HR status, gBRCAm status, site of metastasis, stage of disease progression, prior chemotherapy exposure for metastatic BC, or prior platinum-based chemotherapy exposure for BC [79]. Furthermore, significant improvements in patient-reported quality of life, with a greater delay in time to clinically meaningful deterioration, were reported by patients treated with a PARP inhibitor versus TPC in both OlympiAD and EMBRCA [60, 65]. The Phase IIIb LUCY trial assessed the effectiveness and safety of olaparib in a population of patients with gBRCAm, HER2-negative, metastatic BC that reflected a clinical practice setting [76]. No new safety signals were reported with olaparib in this close-to-real-world setting [76]. Median investigator-assessed PFS and median OS exceeded survival outcomes in OlympiAD, and median OS was longer in patients who received first-line olaparib than in second- or third-line settings, reaffirming OlympiAD findings [76].

**Table 2:** Selected clinical trials of PARP inhibitors and platinum-based chemotherapy for locally advanced or metastatic gBRCAm BC.

Treatments	Clinical trial name /identifier	Study design	Phase	BC patient population	N	Selected efficacy endpoints and outcomes
Olaparib						
Olaparib vs TPC	OlympiAD/ NCT02000622 [60–63,67]	R, OL	III	gBRCAm HER2-negative Metastatic	302	Median PFS (primary endpoint) favoured olaparib vs TPC: 7.0 vs 4.2 months (hazard ratio 0.58 [95% CI 0.43, 0.80]; *P* < 0.001) Median OS: no significant difference vs TPC: 19.3 vs 17.1 months (hazard ratio 0.90 [95% CI 0.66, 1.23]; *P* = 0.513) ORR: favoured olaparib vs TPC: 59.9% (95% CI 52.0, 67.4) vs 28.8% (95% CI 18.3, 41.3) PROs: favoured olaparib vs TPC: mean (standard deviation) change in EORTC QLQ-C30 two-item global QoL scale score: 3.9 (1.2) vs −3.6 (2.2); *P* = 0.0035 Best overall response of ‘improvement’ in global health status/QoL: 33.7% vs 13.4% Subgroup analysis in Asian patients: median PFS favoured olaparib vs TPC: 5.7 vs 4.2 months (hazard ratio 0.53 [95% CI 0.29, 0.97])
Olaparib	LUCY/ NCT03286842 [76]	OL	IIIb	gBRCAm or sBRCAm HER2-negative Metastatic	255	gBRCAm cohort (*n* = 252): Median PFS 8.2 months (95% CI 7.0, 9.2) Median OS 24.9 months (95% CI 21.1, 28.9) Subgroup analysis: median OS longest in participants with HR-positive metastatic BC and in those who received olaparib as first-line therapy for metastatic disease is 27.4 months (95% CI 22.7, 33.5) in HR-positive vs 21.1 months (95% CI 17.1, 27.3) in TNBC, and 27.4 months (95% CI 21.4, 37.4) with first-line olaparib vs 22.7 months (95% CI 18.0, 27.2) with second-/third-line olaparib
Olaparib	Olaparib Expanded/ NCT03344965 [68]	OL	II	HER2-negative or HER2-positive Metastatic Cohort 1: germline mutations in non-BRCA DDR genes Cohort 2: somatic mutations in non-BRCA DDR genes or sBRCAm, with no gBRCAm	54	ORR (primary endpoint) is similar between cohorts: Cohort 1: 33% (95% CI 19, 51), *n* = 9/27 Cohort 2: 31% (95% CI 15, 49), *n* = 8/26 Median PFS (secondary endpoint) Cohort 1: 4.5 months (90% CI 1.7, 12) Cohort 2: 4.1 months (90% CI 2.8, 6.3)
Talazoparib						
Talazoparib vs TPC	EMBRACA/ NCT01945775 [64–66]	R, OL	III	gBRCAm HER2-negative Locally advanced or metastatic	431	Median PFS (primary endpoint) favoured talazoparib vs TPC: 8.6 vs 5.6 months (hazard ratio 0.54 [95% CI 0.41, 0.71]; *P* < 0.001) Median OS – no significant difference vs TPC: 19.3 vs 19.5 months (hazard ratio 0.85 [95% CI 0.67, 1.07]; *P* = 0.17) ORR – favoured talazoparib vs TPC: 62.6% vs 27.2% (odds ratio 5.0 [95% CI 2.9, 8.8]; *P* < 0.001) PROs – favoured talazoparib vs TPC: mean change in EORTC QLQ-C30 two-item global QoL scale score: 3.0 (95% CI 1.2, 4.8) vs −5.4 (95% CI − 8.8 to −2.0); *P* < 0.0001
Talazoparib	ABRAZO/ NCT02034916 [69]	OL	II	gBRCAm HER2-negative or HER2-positive Metastatic Cohort 1: platinum-sensitive Cohort 2: heavily pre-treated (≥3 prior therapies) and no prior platinum	84	ORR (primary endpoint): Overall: 28% (CR, *n* = 2; PR, *n* = 21) Cohort 1: 21% (CR, *n* = 2; PR, *n* = 8) Cohort 2: 37% (CR, *n* = 0; PR, *n* = 13) Median DoR: Overall: 4.9 months Cohort 1: 5.8 months Cohort 2: 3.8 months CBR: Overall: 35% Cohort 1: 27% Cohort 2: 46% ORR in specific subgroups: *BRCA1*m, 23%; TNBC, 26%; HR-positive, 29%; *BRCA2*m, 33% Median PFS: Cohort 1: 4.0 months (95% CI 2.8, 5.4) Cohort 2: 5.6 months (95% CI 5.5, 7.8) Median OS: Cohort 1: 12.7 months (95% CI 9.6, 15.8) Cohort 2: 14.7 months (95% CI 11.0, 24.4)
Talazoparib	BEYOND BRCA/NCT02401347 [70]	OL	II	Non-gBRCAm HRD HER2-negative Advanced	13	ORR: 31% (95% CI 9, 61) (g*PALB2*m, *n* = 3; g*CHEK2*m/g*FANCA*m/s*PTEN*m, *n* = 1) CBR: 54% (95% CI 21, 81)
Talazoparib	NCT03343054 [71]	OL	I	gBRCAm HER2-negative Locally advanced or metastatic Japanese	19	ORR (primary endpoint): 57.9% (90% CI 36.8, 77.0) Median PFS: 7.2 months (95% CI 4.1, NE) Median OS: not reached 12-month OS: 84.7% (90% CI 57.5, 95.1)
Veliparib						
Veliparib + platinum-based chemotherapy OR placebo + platinum-based chemotherapy	BROCADE3/ NCT02163694 [72]	R, DB, PC	III	gBRCAm HER2-negative Locally advanced (unresectable) or metastatic	509	Median PFS (primary endpoint) favoured veliparib vs placebo: 14.5 vs 12.6 months (hazard ratio 0.71 [95% CI 0.57, 0.88]; *P* = 0.0016)
Pamiparib						
Pamiparib	NCT03575065 [84]	OL	II	gBRCAm HER2-negative Locally advanced or metastatic	88	ORR in specific subgroups TNBC, 38.2% (95% CI 25.4, 52.3); HR-positive, 61.9% (95% CI 38.4, 81.9) DoR in specific subgroups TNBC, 7.0 months (95% CI 3.9, NE); HR-positive, 7.5 months (95% CI 5.6, 14.8)
Platinum-based chemotherapy						
Carboplatin OR docetaxel	TNT/ NCT00532727 [73]	R, OL, C, PG	III	TNBC Advanced	376	ORR (primary endpoint): no difference between carboplatin and docetaxel: 31.4% (*n* = 59/188) vs 34.0% (*n* = 64/188); *P* = 0.66 Median PFS: no difference between carboplatin and docetaxel: 3.1 (95% CI 2.4, 4.2) vs 4.4 months (95% CI 4.1, 5.1); *P* = 0.40 Median OS: no difference between carboplatin and docetaxel:12.8 (95% CI 10.6, 15.3) vs 12.0 months (95% CI 10.2, 13.0); *P* = 0.96
Cisplatin	NCT01611727 [74]	OL	II	g*BRCA1*m Metastatic	20	ORR (primary endpoint): 80% (CR, *n* = 9; PR, *n* = 7) OS: 1 year, 80%; 2 years, 60%; 3 years, 25%
Cisplatin OR carboplatin	NCT00483223 [75]	OL	II	TNBC Metastatic or locally recurrent	86	ORR (coprimary endpoint): Overall 25.6% (95% CI 16.8, 36.0); cisplatin, 32.6% (95% CI 19.1, 48.5); carboplatin, 18.6% (95% CI 8.4, 33.4) Individuals with gBRCAm were more likely to achieve a response than those with non-gBRCAm: 54.5% (95% CI 23.4, 83.3) vs 19.7% (95% CI 10.9, 31.3); *P* = 0.022

*BC* breast cancer, *C* controlled, *CBR* clinical benefit rate, *CI* confidence interval, *CR* complete response, *DB* double-blind, *DDR* DNA damage response, *DoR* duration of response, *EORTC QLQ-C30* European Organisation for Research and Treatment of Cancer Quality of Life Questionnaire Core 30-item module, *gBRCAm* germline breast cancer gene mutation, *HER2* human epidermal growth factor receptor 2, *HR* hormone receptor, *HRD* homologous recombination deficiency, *NE* not estimable, *OL* open-label, *ORR* objective response rate, *OS* overall survival, *PARP* poly(ADP-ribose) polymerase, *QoL* quality of life, *PC* placebo-controlled, *PFS* progression-free survival, *PG* parallel-group, *PR* partial response, *PRO* patient-reported outcome, *QoL* quality of life*, R* randomised*, sBRCAm* somatic breast cancer gene mutation, *TNBC* triple-negative breast cancer, *TPC* single-agent standard therapy of the physician’s choice.

There are currently no head-to-head comparisons assessing the efficacy and safety of olaparib versus talazoparib. However, data from OlympiAD and EMBRACA were compared indirectly using fixed-effects and random-effects Bayesian modelling [80, 81]. Both models suggested comparable PFS with olaparib or talazoparib monotherapy, while olaparib was associated with a reduced incidence of alopecia, fewer haematological adverse events (including anaemia, thrombocytopenia and neutropenia), and a greater likelihood of nausea and vomiting relative to talazoparib [80, 81]. However, these findings may be limited by differences in study design and the method used for adverse event reporting [60, 64, 80, 81].

Encouraging findings were also reported from the Phase III BROCADE3 trial of veliparib versus placebo, in combination with carboplatin–paclitaxel, in patients with gBRCAm, HER2-negative, locally advanced, or metastatic BC. Veliparib improved median PFS compared with placebo (Table 2), although serious adverse events were common [72]. The delayed separation of PFS curves was considered related to a subset of patients who discontinued carboplatin–paclitaxel before disease progression and continued on blinded monotherapy; many of these patients also received an increased dose of monotherapy (escalated from 300 to 400 mg twice daily) [72]. Median PFS for veliparib was significantly longer in a subgroup of patients in BROCADE3 who had not received prior cytotoxic therapy for metastatic disease than in the control group, suggesting benefit when veliparib is used in earlier lines of treatment for advanced disease [82]. Exploratory analysis of BROCADE3 also showed that veliparib maintenance monotherapy has a tolerable safety profile and may extend PFS [83].

The investigational PARP inhibitor pamiparib has shown an encouraging efficacy and safety in a Phase II trial of patients with TNBC and HR-positive/HER2-negative gBRCAm, with a trend towards higher objective response rate (ORR) in patients who had received fewer prior lines of chemotherapy, or those who were platinum naïve [84].

#### Platinum-based chemotherapy

International guidelines include platinum agents (cisplatin and carboplatin) as a preferred treatment option for patients with recurrent unresectable or metastatic gBRCAm TNBC, although it is not yet clear how they compare with PARP inhibitors in this setting [47]. Findings from the TNT Phase III study suggest that gBRCAm may predict a favourable response to carboplatin monotherapy compared with docetaxel [73]. While there were no significant differences in ORR, median PFS, or median OS between the two treatment arms in the overall population, significant improvements in ORR and PFS were reported with carboplatin versus docetaxel in the subgroup of patients with gBRCAm TNBC [73]. Promising results have also been reported for platinum-based chemotherapy in Phase II trials, albeit in small numbers of patients with gBRCAm metastatic BC (Table 2) [74, 75].

#### Cyclin-dependant kinase 4/6 inhibitors

A growing body of real-world data suggests that patients with gBRCAm may have suboptimal BC treatment outcomes with CDK4/6 inhibitors. For example, analysis of 2968 patients in the US Flatiron Health database revealed a shorter time to first subsequent therapy or death and shorter OS in individuals with gBRCAm HR-positive/HER2-negative metastatic BC compared with patients with non-gBRCAm disease [5]. Similarly, two retrospective analysis of real-world data from patients with HR-positive, HER2-negative metastatic BC treated with CDK4/6 inhibitors plus endocrine therapy found that germline pathogenic variants in DDR-related genes (including *BRCA1*, *BRCA2*, *ATM* and *CHEK2*) were independent prognostic factors for shorter PFS and OS [85, 86]. In addition, exploratory analysis of the Phase III PADA-1 study reported that patients with HR-positive/HER2-negative metastatic BC and germline pathogenic variants in *BRCA1*, *BRCA2* or *PALB2* had shorter PFS than non-carriers following endocrine therapy and palbociclib [87]. These observations are consistent with a retrospective analyses of genomic data, in which g*BRCA1*m was identified among the alterations associated with CDK4/6 inhibitor resistance in patients with HR-positive, metastatic BC [88], and g*BRCA2*m was associated with shorter PFS in patients with BC who received CDK4/6 inhibitors plus endocrine therapy [89]. A strong association was identified between g*BRCA2*m and pathogenic somatic *RB1* alterations [89], which are known drivers of resistance to CDK4/6 inhibitors [90, 91].

### Current innovation and future advances in the treatment of gBRCAm, HER2-negative, early, locally advanced or metastatic breast cancer

#### PARP inhibitor combinations

To overcome the development of resistance and increase sensitivity to PARP inhibitors, several studies are investigating combinations with other drug classes [92]. For advanced gBRCAm BC, Phase I/II clinical trials have demonstrated positive outcomes when olaparib or niraparib were combined with programmed cell death 1 (PD-1) or programmed cell death ligand 1 (PD-L1) immune checkpoint inhibitors [93, 94]. Olaparib plus durvalumab demonstrated a 12-week disease control rate of 80% in patients with gBRCAm metastatic BC in the MEDIOLA trial, although the trial did not include an olaparib-only arm for comparison [93]. In the TOPACIO trial, niraparib plus pembrolizumab was associated with an ORR of 21% and a disease control rate of 49% in the overall advanced or metastatic TNBC population, which increased to 47% and 80% in the gBRCAm cohort [94].

A Phase II study of 78 patients with HER2-negative, BRCAm, advanced or metastatic BC (NCT02849496) reported that addition of atezolizumab did not significantly improve PFS, compared with olaparib monotherapy. However, this small study included patients that had received prior hormone therapy and chemotherapy, and patients were not stratified by PD-L1 expression [95]. PD-L1 expression has been shown to be associated with clinical benefit with PD-1/PD-L1 checkpoint inhibitor treatment in metastatic TNBC [96], and may therefore be a useful biomarker for predicting response to PARP inhibitor/immune checkpoint inhibitor combinations, although this requires further investigation. The KEYLYNK-009 trial investigated olaparib in combination with pembrolizumab in patients with metastatic TNBC. Numerical improvements in PFS and OS were observed in patients with TNBC and tumour *BRCA1* and/or *BRCA2* mutations (tBRCAm) who received olaparib plus pembrolizumab versus pembrolizumab plus chemotherapy. However, no survival benefit was reported in the unselected study population [97].

Clinical trials are building on promising results with PARP inhibitor and immune checkpoint inhibitor combinations in the early BC setting. The OlympiaN trial is adopting a risk-based approach to neoadjuvant treatment for patients with oestrogen receptor-negative or -low, HER2-negative, BRCAm BC. Patients with a lower tumour burden (T1b-c/N) are assigned olaparib monotherapy, and those with a greater risk of recurrence determined by tumour burden (T2/N0 or T1/N1) are assigned olaparib in combination with durvalumab. Other clinical trials are evaluating the combination of olaparib and pembrolizumab as neoadjuvant therapy (NCT05203445), or as adjuvant therapy after neoadjuvant pembrolizumab plus paclitaxel and carboplatin (NCT05485766). A Phase II clinical trial is also evaluating niraparib with dostarlimab as neoadjuvant treatment for patients with BRCAm BC (NCT04584255).

Although PARP inhibitor and immune checkpoint inhibitor combinations look promising, other investigational PARP inhibitor combinations have been met with mixed success. For example, olaparib in combination with the WEE1 and ATR inhibitors adavosertib and ceralasertib as second- or third-line therapy for metastatic TNBC did not improve PFS versus single-agent olaparib in the Phase II VIOLETTE study either in the overall (primary endpoint) or BRCAm populations [98]. Olaparib in combination with the AKT inhibitor capivasertib was more promising in a Phase I trial, demonstrating clinical benefit (complete response, partial response, or stable disease ≥4 months) in 44.4% of the overall advanced BC population, and in 71.4% of the gBRCAm BC cohort [99].

#### Treatment sequencing

The recommended first-line therapy for certain patients with HER2-negative, recurrent unresectable or metastatic, gBRCAm BC is a PARP inhibitor (olaparib or talazoparib) [47]. However, how should treating physicians adapt their practice in light of the recent regulatory approval of olaparib in the adjuvant setting? More research is needed, but some evidence can be gleaned from clinical trials with PARP inhibitors in other tumour types. For example, the Phase III OReO/ENGOT Ov-38 trial showed that rechallenge with maintenance olaparib in patients with platinum-based chemotherapy-sensitive ovarian cancer who were previously treated with a PARP inhibitor resulted in longer PFS compared with placebo [100]. Although these findings cannot be used to infer similar benefits in PARP inhibitor rechallenge in BC, they set a precedent for future BC trials to address this outstanding question. In the absence of rechallenge data, studies have sought to investigate treatment sequencing in relation to PARP inhibitors and platinum-based chemotherapy; evidence of activity has been shown with PARP inhibitors in patients who had progressed on previous platinum-based chemotherapy [4]. Furthermore, a single-institution study of real-world data from patients treated for BRCAm, advanced BC showed that PFS was improved in patients who received a first-line PARP inhibitor but was worse in patients who received a PARP inhibitor after platinum-based chemotherapy [101].

#### Beyond gBRCAm

Genetic abnormalities other than gBRCAm can also cause HRD, providing rationale for using targeted therapies in patients with these mutations [77]. PARP inhibitor combination therapy is approved for HRD-positive advanced ovarian cancer, identified through BRCAm and genomic instability testing [29, 30]. Olaparib can also be offered as maintenance treatment for patients with gBRCAm or somatic BRCAm (sBRCAm) advanced ovarian cancer and in later lines of treatment for gBRCAm advanced ovarian cancer [29]. Indeed, a meta-analysis of PARP inhibitor studies including patients with gBRCAm or sBRCAm revealed comparable ORR for these alterations [102]. Subgroup analyses showed no difference between tumour types (ovarian, prostate and pancreatic cancer) or PARP inhibitors (olaparib, rucaparib, or niraparib), supporting wider consideration for treating sBRCAm tumours with these agents [102]. However, limited studies have examined the efficacy of PARP inhibitors and platinum-based chemotherapy for sBRCAm and non-BRCAm HRD-positive BC. No significant association between HRD score and residual cancer burden response to cisplatin was seen in the Phase II TBCRC 030 study [103], whereas the Phase II TBCRC 048 study reported a significantly higher ORR and longer PFS with olaparib in patients with germline *PALB2* mutations compared with patients with sBRCAm [68]. The RUBY trial, investigating rucaparib in HER2-negative, metastatic BC, suggested that a subset of patients without gBRCAm, but with other genomic instabilities, may benefit from PARP inhibitor treatment [104]. In the Phase II PETREMAC study, responses were seen with olaparib in patients with TNBC and various homologous recombination repair gene mutations [105], while subsequent analysis of gene panel sequencing data established criteria to accurately detect HRD, which may be used to identify a wider subset of patients with multiple BC subtypes who are likely to respond to HRD-targeted therapy [106].

## gBRCAm testing in breast cancer

### The importance of early detection of gBRCAm

Early detection of patients with gBRCAm allows oncologists to recommend the most suitable treatment pathway for their patients to achieve the best outcomes. Knowledge of mutation status is critical in guiding surgical options to select the most risk-reducing approach, can inform the choice of chemotherapy (platinum vs taxane) in the metastatic setting, and can allow prompt initiation of PARP inhibitor treatment, where indicated [6, 25, 34, 47, 57, 60, 64, 73]. Early gBRCAm detection also enables at-risk family members to be offered monitoring, screening or risk-reducing measures.

### Who to test for gBRCAm

International guidelines have set out recommendations for who should be referred to genetic testing (Table 3). Encompassing early BC, the NCCN Guidelines^®^ recommend gBRCAm testing for all individuals who receive BC diagnoses at an early age (≤50 years) and for those diagnosed with TNBC, irrespective of their age, to aid in adjuvant treatment decisions with olaparib for high-risk, HER2-negative BC [25]. ESMO 2023 guidelines recommend gBRCA testing in patients with early BC who meet the respective national criteria for germline testing and in those who are candidates for adjuvant olaparib therapy [48]. For patients with metastatic BC, NCCN Guidelines recommend that all patients are assessed for gBRCAm to identify candidates for PARP inhibitor therapy [25], while ESMO 2023 guidelines recommend gBRCA testing in all patients at first diagnosis of HER-2-negative metastatic BC (Table 3) [107]. In addition to international BRCA testing guidelines, further guidance may be provided at a national level. For example, the National Genomic Test Directory specifies testing criteria for the most appropriate publicly funded genetic tests available through the National Health Service in England (NHS England) [108].

**Table 3:** Criteria recommended by the NCCN, ESMO and ASCO-SSO to assess eligibility for gBRCAm testing for individuals with a personal history of BC.^a^

NCCN Clinical Practice Guidelines in Oncology (NCCN Guidelines^®^) (all settings)^b^ [25]
• Any patient diagnosed with BC ≤ 50 years of age • At any age: ∘ TNBC diagnosis ∘ **Early BC**: to aid in adjuvant treatment decisions with olaparib in high-risk, HER2-negative BC ∘ **Metastatic BC**: to aid in systemic treatment decisions using PARP inhibitors for BC ∘ Multiple primary BCs (synchronous or metachronous) or lobular BC with personal/family history of diffuse gastric cancer ∘ Male BC ∘ Ashkenazi Jewish ancestry ∘ Based on family history: ▪ ≥1 close relative(s) with BC ≤ 50 years of age, male BC, ovarian, pancreatic or prostate cancer (metastatic, or high/very high-risk group) ▪ ≥3 diagnoses of BC and/or prostate cancer (any grade) on the same side of the family, including the patient with BC
**ESMO 2023 guideline recommendations** [48, 107]
• Patients with **early BC**: ∘ Who are candidates for adjuvant olaparib therapy ∘ Who meet national criteria for germline testing • Patients with **metastatic BC**: ∘ At first diagnosis of HER2-negative metastatic BC ∘ Following failure of CDK4/6 inhibitor therapy in ER-positive, HER2-negative metastatic BC
**ASCO–SSO 2024 guideline recommendations** [111]
• Patients diagnosed with breast cancer ≤65 years of age • Patients >65 years of age diagnosed with breast cancer if: ∘ They are candidates for PARP inhibitor therapy for early-stage or metastatic disease ∘ They have TNBC ∘ Their personal or family history suggests the possibility of a pathogenic variant ∘ They were assigned male sex at birth ∘ They are of Ashkenazi Jewish ancestry or are members of a population with an increased prevalence of founder mutations • At any age: ∘ Patients with a second primary cancer in the contralateral or ipsilateral breast ∘ Patients with recurrent BC (local or metastatic), who are candidates for PARPi therapy, regardless of family history

*ASCO–SSO* American Society of Clinical Oncology and Society of Surgical Oncology, *BC* breast cancer, *CDK4/6* cyclin-dependent kinase 4/6, *ER* oestrogen receptor, *ESMO* European Society for Medical Oncology, *gBRCAm* germline breast cancer gene mutation, *HER2* human epidermal growth factor receptor 2, *NCCN* National Comprehensive Cancer Network^®^ (NCCN^®^), *PARP* poly(ADP-ribose) polymerase, *TNBC* triple-negative breast cancer.

^a^Individuals may have one or more of the listed criteria, in addition to a personal history of BC.

^b^Given the high prevalence of founder gBRCAm in patients with Ashkenazi Jewish heritage, NCCN Guidelines suggest that universal testing for founder BRCA1/2 pathogenic/likely pathogenic variants in individuals of Ashkenazi Jewish ancestry, regardless of personal or family history, should be offered primarily in the setting of longitudinal research studies. If there is no access to longitudinal studies, then testing may be offered when pre- and post-test genetic counselling is available.

Previous studies of gBRCA testing indicated that a non-negligible proportion of patients with gBRCAm breast cancer were not eligible for gBRCA testing per treatment guidelines [8, 109, 110], and concerns about the underutilisation of testing have led to re-evaluation of current genetic testing criteria. If a patient (<60 years of age) with personal history of BC does not meet testing criteria, NCCN Guidelines suggest that testing can be considered alongside genetic counselling [25]. The American Society of Clinical Oncology and Society of Surgical Oncology (ASCO–SSO) recommend gBRCA testing in newly diagnosed BC patients aged 65 years or younger [111], while others have proposed that that all women up to 60 years of age diagnosed with BC undergo testing [112], and the American Society of Breast Surgeons has advocated testing for all patients diagnosed with BC, regardless of age [113].

Both NCCN Guidelines and ASCO-SSO recommend gBRCAm testing in certain groups based on personal history, family history, pathology, histology, ancestry, or eligibility for PARP inhibitor therapy (Table 3) [25, 111]. It is noteworthy that NCCN Guidelines recommend BRCA testing in individuals of Ashkenazi Jewish ancestry who have personal history of breast cancer, due to the high prevalence of founder gBRCAm among these individuals [25]. Although a family history of certain cancers (including non-BC) can also be a criterion for testing, this may be unknown to some patients and should not preclude eligibility for gBRCAm testing if other criteria are met. However, evidence suggests that patients with an unknown family history are more likely to undergo BRCA testing if they have TNBC compared with HR-positive/HER2-negative disease [114]. For women with no personal history of BRCAm cancer but with family history of BRCA-associated malignancies, the United States Preventive Services Task Force (USPSTF) recommends that primary care clinicians screen patients for referral to genetic counselling and potential genetic testing [115]. They conclude that the net benefit of risk assessment for an increased risk of BRCAm, BRCA testing, and use of risk-reducing interventions outweighs any potential harm in women whose family or personal history is associated with an increased risk for potentially harmful BRCAm [115].

### How to test for gBRCAm

BRCAm testing is generally based on next-generation sequencing of patient DNA. Targeted sequencing is used to analyse a particular gene(s) of interest, and is the cheapest, most accurate and easiest to interpret. The BRACAnalysis CDx test (Myriad Genetics, Salt Lake City, UT, USA) is the only companion gBRCAm diagnostic test approved by the US Food and Drug Administration to evaluate olaparib suitability in BC [116]. In several other countries, the companion gBRCAm diagnostic test for olaparib is not specified. Gene panel testing allows for the analysis of multiple genes beyond *BRCA1* and *BRCA2* that may also be associated with tumour development and/or treatment response, and their feasibility for guiding BC treatment is being considered in clinical trials [68, 70, 105]. Whole exome and whole genome sequencing provide more comprehensive characterisation of patient DNA and can be used to uncover novel variants associated with a particular disease or condition. However these methods can be expensive and time-consuming and are generally confined to the research setting [117, 118].

### Overcoming the challenges of undertesting for gBRCAm

Critical to the success of testing strategies for gBRCAm is the availability of affordable genetic tests with fast turnaround times. It is important to ensure that all eligible patients are referred for fast-track genotyping and associated counselling processes [6], and that uptake of testing by patients is as high as possible. Referral rates for genetic testing in BC remain suboptimal, despite guideline recommendations[119]. For example, a 2017 US survey reported that only 29% of patients who met genetic testing eligibility criteria discussed testing with a healthcare practitioner, and only 15% underwent genetic testing [7]. A recent retrospective study analysed data from 3672 patients who had received a diagnosis of HR-positive/HER2-negative early BC and a confirmed Oncotype DX test. Although an increasing trend in *g*BRCA testing was observed over time, only one third of patients diagnosed between 2011 and 2022 underwent BRCAm testing [120]. A mandate from a large US national healthcare insurance payer that required patients to consult with a geneticist or certified genetic counsellor before genetic testing resulted in increases in test cancellations [121]. BRCAm test cancellations between the 12 months prior to and the 12 months after the mandate date significantly increased among all insured patients (*P* < 0.001) and in the subgroup of patients meeting NCCN testing criteria (*P* < 0.001) [121]. Strategies to increase uptake of BRCAm testing among patients include better dissemination of information to high-risk individuals, free genetic counselling, provision of an immediate option for testing after video-assisted counselling, and discussion of how individuals can inform their family of the diagnosis [122, 123].

In the USA, gBRCAm is reported in 11% of patients with BC who have no family history of BC or ovarian cancer [124], and ~20% of patients with gBRCAm are reported not to meet NCCN germline testing criteria [8]. To overcome the barrier of ineligibility based on familial and risk-based criteria, population-based screening can be used to identify individuals at high risk of BRCAm [125]. For example, the Israeli Ministry of Health and NHS England support gBRCA testing for individuals with Jewish ancestry, irrespective of personal history of cancer, and the USPSTF recommend risk assessment in unaffected individuals who have ancestry associated with increased gBRCAm prevalence [115, 125, 126]. Screening for BRCAm and other DDR gene mutations in the USA and the UK could potentially reduce BC cases by 1.9% and prevent 367 and 523 deaths per million women in each country, respectively [127]. Screening could potentially be implemented during routine care, e.g. mammogram appointments, to increase genetic referral rates [128]. The cost-effectiveness of population-based genomic screening for hereditary BC and ovarian cancer in unselected women may depend on the age of the individuals screened: screening of 30-year old women was found to be moderately cost-effective, while screening of 45-year old women was not considered cost-effective [129].

Given that screening would increase the number of individuals identified as eligible for BRCAm testing, strategies are needed to ease the burden on already overstretched genetic counselling services. Adoption of innovative genetic services models could be considered, such as oncologist-led mainstreaming, where genetic testing is arranged by the treating cancer team, with genetic services involved only if a mutation is detected [130]. Other approaches to reduce the burden on genetic counsellors may involve pre-test use of a video, or real-time teleconferencing, followed by face-to-face genetic counselling in the event of a positive test [122, 131, 132]. Novel delivery methods such as web-based systems, chatbots and artificial intelligence risk assessment tools are also options, although they provide less emotional support and may not be appropriate for all situations or patients [131, 133, 134]. Access to genetic testing may be hampered by gaps in healthcare practitioners’ knowledge of BRCAm prevalence and of appropriate screening, testing and interpretation of results [135]. Ongoing provision of training and education for healthcare providers who are involved in mainstream testing is critical to ensure that they are appropriately upskilled to deliver pre-test counselling and obtain consent, and training materials aimed at increasing genetic literacy among healthcare professionals have been developed [136, 137]. Increased awareness is also needed around the challenges and barriers to genetic testing from the perspective of the patient. A number of patient support resources have been generated by advocacy groups [138], and patient-led initiatives are aiming to drive policy changes, improve the patient experience, and ultimately increase uptake of genetic testing [139].

## Conclusions

BRCAm predisposes an individual to develop BC, and BRCAm is associated with an aggressive disease phenotype. The presence of BRCAm helps to guide on preferred options for surgery and systemic treatment, including PARP inhibitors in the adjuvant and metastatic settings. Consequently, timely genetic testing is imperative to enable prompt identification of patients with gBRCAm and ensure appropriate management of their disease. Indeed, available evidence suggests that PARP inhibitors are more effective when used in earlier treatment lines. Despite international guidelines outlining eligibility criteria for genetic testing, there is a clear need to improve referral and uptake rates. Some guideline groups have gone so far as to recommend gBRCAm testing for all patients diagnosed with BC regardless of their age. Careful consideration is warranted to determine how to efficiently identify patients with gBRCAm early in the disease course and provide them with the best available treatment.
